# Elderly Male With Cardiovascular-Related Comorbidities Has a Higher Rate of Fatal Outcomes: A Retrospective Study in 602 Patients With Coronavirus Disease 2019

**DOI:** 10.3389/fcvm.2021.680604

**Published:** 2021-06-07

**Authors:** Xiao-Yong Zhan, Liang Li, Yuhai Hu, Qiang Li, Huimin Kong, Margaret H. L. Ng, Chun Chen, Yulong He, Bihui Huang, Mo Yang

**Affiliations:** ^1^The Seventh Affiliated Hospital, Sun Yat-sen University, Shenzhen, China; ^2^Department of Clinical Laboratory, Hankou Hospital, Wuhan, China; ^3^Department of Clinical Laboratory, Nanfang Hospital, Southern Medical University, Guangzhou, China; ^4^Department of Anatomical and Cellular Pathology, The Chinese University of Hong Kong, Hong Kong, China

**Keywords:** COVID-19, cardiovascular-related comorbidities, aggressive inflammatory response, lymphopenia, elderly male

## Abstract

Elderly with comorbidities have shown a higher rate of fatal outcomes when suffering coronavirus disease 2019 (COVID-19). However, a delineation of clinical significances of hematologic indices and underlying comorbidities in the progression and outcome of COVID-19 remains undefined. Six hundred two COVID-19 patients with established clinical outcomes (discharged or deceased) from Hankou Hospital of Wuhan, China between January 14, 2020 and February 29, 2020 were retrospectively analyzed. Of the 602 patients with COVID-19, 539 were discharged and 63 died in the hospital. The deceased group showed higher leukocyte and neutrophil counts but lower lymphocyte and platelet counts. Longer activated partial thromboplastin time (APTT) and prothrombin time (PT), as well as higher D-dimer and C-reactive protein levels, were found in non-survivors. Our observations suggest that these parameters could serve as potential predictors for the fatal outcome and in the discharged group. A higher neutrophil count and D-dimer level but lower lymphocyte were associated with a longer duration of hospitalization. A multivariable Cox regression analysis showed that higher neutrophil count, prolonged PT, and low lymphocyte count were risk factors for patients with COVID-19. Also, we found an association of lower lymphocyte count and higher C-reactive protein levels with the elderly group and those with cardiovascular-related comorbidities. The significantly different hematologic profiles between survivors and non-survivors support that distinct hematologic signatures in COVID-19 patients will dictate different outcomes as a prognostic marker for recovery or fatality. Lymphopenia and aggressive inflammatory response might be major causes for fatal outcomes in the elderly male and especially those with cardiovascular-related comorbidities.

## Introduction

Severe acute respiratory syndrome coronavirus 2 (SARS-CoV-2) emerged and is now a worldwide health threat (1). Up to December 2020, tens of thousands of patients are still diagnosed with the coronavirus disease 2019 (COVID-19) every day all over the world. Typically, affected individuals display a variable extent of dyspnea and radiological signs (2–4). Through the unremitting efforts of researchers, we have a deeper understanding of COVID-19. Clinical studies have detected a cytokine storm in critical patients with COVID-19 (5), which is considered to be one of the major causes of acute respiratory distress syndrome (ARDS) and multiple-organ failure at the beginning of the SARS-COV-2 outbreak (6). Thrombotic complications in patients with COVID-19 are common and contribute to organ failure and mortality (7, 8), which suggests that platelet hyperreactivity is associated with SARS-CoV-2 infection and participating in COVID-19 pathophysiology (9). Several reports have described significant procoagulant events, including life-threatening pulmonary embolism (PE) (10–12). According to the last available sex-related study from Italy, lethality is 17.7% in men and 10.8% in women, suggesting gender might also be a risk factor for COVID-19 patients (13, 14). Although many clinical studies have been done on COVID-19, laboratory indices to predict disease progression and prognosis are not well-established yet (15).

Hematological findings and thrombocytopenia with SARS and COVID-19 have been reported in our previous publications (15, 16). In the present study, we present a retrospective analysis to describe clinical outcomes, underlying comorbidities, and hematological indices in 602 laboratory-confirmed hospitalized COVID-19 patients. We aimed to explore the potential factors that predict the prognosis and survival outcome of COVID-19 inpatients. With multiple analyses of bio-indices among patients with different underlying comorbidities or age and gender, we sought to delineate how underlying comorbidities, age, and gender influence the disease outcome.

## Methods

### Ethical Statement

The study was approved by the Ethics Committee of the Seventh Affiliated Hospital, Sun Yat-sen University. No informed consent of patients was required.

### Data Sources

We obtained the medical records of 602 hospitalized patients with a laboratory-confirmed diagnosis of COVID-19 from Wuhan Hospital between January 14, 2020, and February 29, 2020. Admission criteria are as follows: the patient has clinical symptoms, a positive nucleic acid test, and CT suggests viral pneumonia. Demographic information, medical history, comorbidities, signs and symptoms, and laboratory findings on admission were collected from electronic medical records.

A laboratory-confirmed case of COVID-19 was defined as a positive real-time reverse transcriptase–polymerase chain reaction (RT-qPCR) test result obtained through oral pharyngeal swab specimens. Investigators collected demographic information, exposure history, medical history, comorbidities, signs and symptoms, chest computed tomography, laboratory findings on admission, and clinical outcomes from electronic medical records. Laboratory results (blood count, biochemical analysis, and coagulation testing) were included in laboratory profile testing. The dates of disease onset, SARS-CoV-2 laboratory confirmation, hospital admission, discharge, and death were also recorded.

### Statistical Analysis

Continuous variables are presented as medians with interquartile ranges (IQRs). For categorical variables, we calculated the frequency rates and percentages of patients in each category. Continuous variables were compared using the Mann–Whitney *U*-test. Proportions for categorical variables were compared using the chi-square test, and Fisher's exact test was used when the data were limited. *P* < 0.05 was considered statistically significant. Spearman's correlation analysis was used to analyze the relationship between different indices. Survival curves were estimated by the Kaplan–Meier method and compared by the log-rank test. Multivariate Cox regression was performed to investigate the hazard ratio by the Cox proportional hazards model. GraphPad Prism 8 (GraphPad Software) was used for graphing. Statistical analyses were performed using SPSS 25.0 (IBM software). A principal component analysis (PCA) for hematologic indices was performed using Origin (OriginLab).

## Results

### Baseline Clinical Characteristics

A total of 602 patients (383 males and 389 females) with a laboratory-confirmed diagnosis of COVID-19 were included, in which 539 were discharged and 63 died in hospital. The baseline characteristics of these patients are shown in Table 1. The median age for all patients was 62 years, with significantly older age for deceased than for discharged (71.0 vs. 61.0 years; *P* < 0.0001). Among all deceased patients, 65.08% were males, which was significantly more than females (*P* = 0.039). There were 386 patients (386/580, 66.55%) (due to some data missing, only 580 patients had underlying comorbidity records) who had at least one underlying comorbidity, of which 24.66% had only cardiovascular-related underlying comorbidities (CRUC) including hypertension, diabetes, coronary heart disease, and cerebrovascular disease; 18.62% had other underlying comorbidities including thyroid nodules, fracture, chronic renal failure, lymphoma, hepatitis B, gallstone, etc.; and 23.28% had at least two types of underlying comorbidities, in which one was CRUC. The incidence of underlying comorbidities was significantly higher in the deceased than in the discharged (*P* < 0.001).

**Table 1:** Baseline characteristics of the recovered patients and patients who died of COVID-19.

**Characteristics**	**All patients(*n* = 602)**	**Discharged (*n* = 539)**	**Deceased (*n* = 63)**	***P*-value**
**Demographic**				
Survival or death rate (%)		89.53	10.47	N/A
Age, years	62.0 (51.0–70.0)	61.0 (50.0–69.0)	71.0 (64.0–79.0)	< 0.0001
**Gender**				
Female, *n* (%)	289 (48.01)	267 (49.54)	22 (34.92)	0.039^*^
Male, *n* (%)	313 (51.99)	272 (50.46)	41 (65.08)	
Hospitalization, days	18 (12–26)	19 (12–27)	7 (3–13)	N/A
**Comorbidity**				
No comorbidity, *n* (%)	194 (33.44)	186 (35.84)	8 (13.11)	<0.001^*^^&^
Generalized vascular disease, *n* (%)	143 (24.66)	121 (32.31)	22 (36.07)	
Other comorbidity, *n* (%)	108 (18.62)	102 (19.65)	6 (9.84)	
Two and more comorbidities, *n* (%)	135 (23.28)	110 (21.20)	25 (40.98)	
**Laboratory findings**				
**Hematologic**				
**Leukocyte count,10**^**9**^**/l**	5.70 (4.30–7.88)	5.40 (4.20–7.20)	11.70 (8.50–15.50)	<0.0001
<4 × 10^9^/l, *n* (%)	95 (17.24)	94 (18.91)	1 (1.85)	<0.001^*^^#^
4–10 × 10^9^/l, *n* (%)	383 (69.51)	363 (73.04)	20 (3.70)	
>10 × 10^9^/l, *n* (%)	73 (13.25)	40 (8.05)	33 (61.11)	
**Neutrophil count, 10**^**9**^**/l**	4.00 (2.70–6.20)	3.80 (2.60–5.40)	10.50 (7.80–13.48)	<0.0001
<1.8 × 10^9^/l, *n* (%)	33 (6.00)	33 (6.65)	0 (0)	<0.001^*^^#^
1.8–6.3 × 10^9^/l, *n* (%)	384 (69.82)	375 (75.60)	9 (16.67)	
>6.3 × 10^9^/l, *n* (%)	133 (24.18)	88 (17.74)	45 (83.33)	
**Lymphocyte count, 10**^**9**^**/l**	1.00 (0.70–1.50)	1.10 (0.70–1.50)	0.5 (0.30–0.70)	<0.0001
<0.8 × 10^9^/l, *n* (%)	169 (30.73)	127 (25.60)	42 (77.78)	<0.001^*^
0.8–4.0 × 10^9^/l, *n* (%)	381 (69.27)	369 (74.40)	12 (22.22)	
**Hemoglobin, g/l**	126.00 (115.00–135.00)	126.00 (115.00–135.00)	131.00 (110.00–139.00)	0.2559
**Platelet count, 10**^**9**^**/l**	221.00 (169.00–290.00)	230.00 (174.00–294.00)	170.00 (78.00–231.30)	<0.0001
<100 × 10^9^/l, *n* (%)	25 (6.19)	14 (3.88)	11 (25.58)	<0.001^*^^$^, 0.117^∧^
100–300 × 10^9^/l, *n* (%)	295 (73.02)	268 (74.24)	27 (62.79)	
>300 × 10^9^/l, *n* (%)	84 (20.79)	79 (21.88)	5 (11.63)	
**Other indices**				
**APTT, s**	34.40 (31.30–37.90)	34.20 (31.30–37.20)	37.00 (31.30–42.70)	0.0152
≤ 47 s, *n* (%)	386 (94.84)	352 (96.97)	34 (72.27)	<0.001^*^
>47 s, *n* (%)	21 (5.16)	11 (3.03)	10 (22.73)	
**Prothrombin time (PT), s**	13.85 (12.93–15.00)	13.70 (12.80–14.70)	17.10 (14.60–19.95)	<0.0001
≤ 17 s, *n* (%)	380 (93.37)	349 (96.14)	21 (47.73)	<0.001^*^
>17 s, *n* (%)	37 (6.63)	14 (3.86)	23 (52.27)	
**Thrombin time (TT), s**	15.70 (15.00–16.60)	15.70 (15.00–16.40)	16.30 (14.85–17.90)	0.0664
≤ 19 s, *n* (%)	393 (96.56)	357 (98.35)	36 (81.82)	<0.001^*^
>19 s, *n* (%)	14 (3.44)	6 (1.65)	8 (18.18)	
**D-dimer, mg/l**	0.65 (0.18–3.02)	0.54 (0.15–2.02)	6.96 (0.95–8.00)	<0.0001
<0.5 mg/l, *n* (%)	181 (44.47)	177 (48.76)	4 (9.09)	<0.001^*^
≥0.5 mg/l, *n* (%)	226 (55.53)	186 (51.24)	40 (90.91)	
**Fibrinogen (FIB), g/l**	3.29 (2.58–4.18)	3.29 (2.59–4.18)	3.06 (1.46–4.41)	0.6432
<2, *n* (%)	35 (8.60)	23 (6.34)	12 (27.27)	<0.001^*^^$^, 0.412^*^^∧^
2–4, *n* (%)	246 (60.44)	230 (63.36)	16 (36.36)	
>4, *n* (%)	126 (30.96)	110 (30.30)	16 (36.36)	
**International normalized ratio (INR)**				
≤ 1.5, *n* (%)	385 (94.59)	355 (97.80)	30 (68.18)	<0.001^*^
>1.5, *n* (%)	22 (5.41)	8 (2.20)	14 (31.82)	
**C-reactive protein (CRP), mg/l**	10.30 (2.08–49.15)	7.97 (1.79–42.20)	115.00 (42.88–199.30)	<0.0001
≤ 10 mg/l, *n* (%)	152 (49.51)	150 (53.38)	2 (7.69)	<0.001^*^
>10 mg/l, *n* (%)	155 (50.49)	131 (46.62)	24 (92.31)	

* *Chi-square tests or Fisher's exact test was used to compare the COVID-19 mortality between the patients with different indices*.

& *Patients with or without comorbidities were compared*.

# *COVID-19 mortality of patients with high neutrophil count (>6.3 × 10^9^/l) or leukocyte count (>6.3 × 10^9^/l) was compared with the other two groups*.

$ *COVID-19 mortality of low-platelet-count group (<100 × 10^9^/L) and low-level FIB group (<2.0 g/l) was compared with the other two groups*.

∧ *COVID-19 mortality of high-platelet-count group (>100 × 10^9^/l) and high-level FIB group (>4.0 g/l) was compared with the other two groups. N/A, not available*.

### Laboratory Findings Among Hospitalized Patients With Different Outcomes

**Figure 1:**
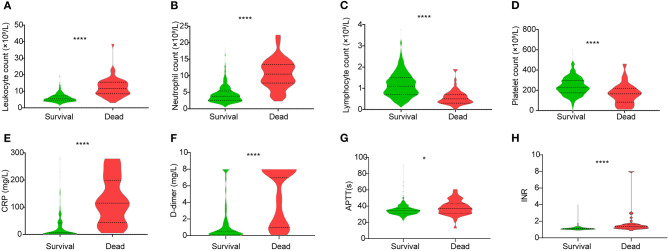
Different levels of hematologic indices **(A)** leukocyte count, **(B)** neutrophil count, **(C)** lymphocyte count, **(D)** platelet count, **(E)** CRP level, **(F)** D-dimer level, **(G)** APTT, and **(H)** INR between the discharged and deceased patients. Data are shown as a violin plot with median and 25 and 75% percentile lines. *P < 0.05, ****P < 0.0001. CRP, C-reactive protein; APTT, activated partial thromboplastin time; INR, international normalized ratio.

The hematologic profile among patients with different outcomes is shown in Table 1. Compared with the discharged, higher leukocyte and neutrophil counts (Figures 1A,B), but lower lymphocyte and platelet counts (Figures 1C,D), were found in deceased patients. Lymphopenia (<1 × 10^9^/l) was more common in non-survivors than survivors (Table 1).

Compared with discharged patients, the deceased also showed significantly increased levels of C-reactive protein (CRP) and D-dimer (Figures 1E,F). Regarding the coagulation indicators, activated partial thromboplastin time (APTT), thrombin time (TT), prothrombin time (PT), and international normalized ratio (INR) were all increased in deceased patients (Figures 1G,H, Table 1).

### Association of Patient Characteristics and Laboratory Indices With the Survival Rate of COVID-19 Patients

**Figure 2:**
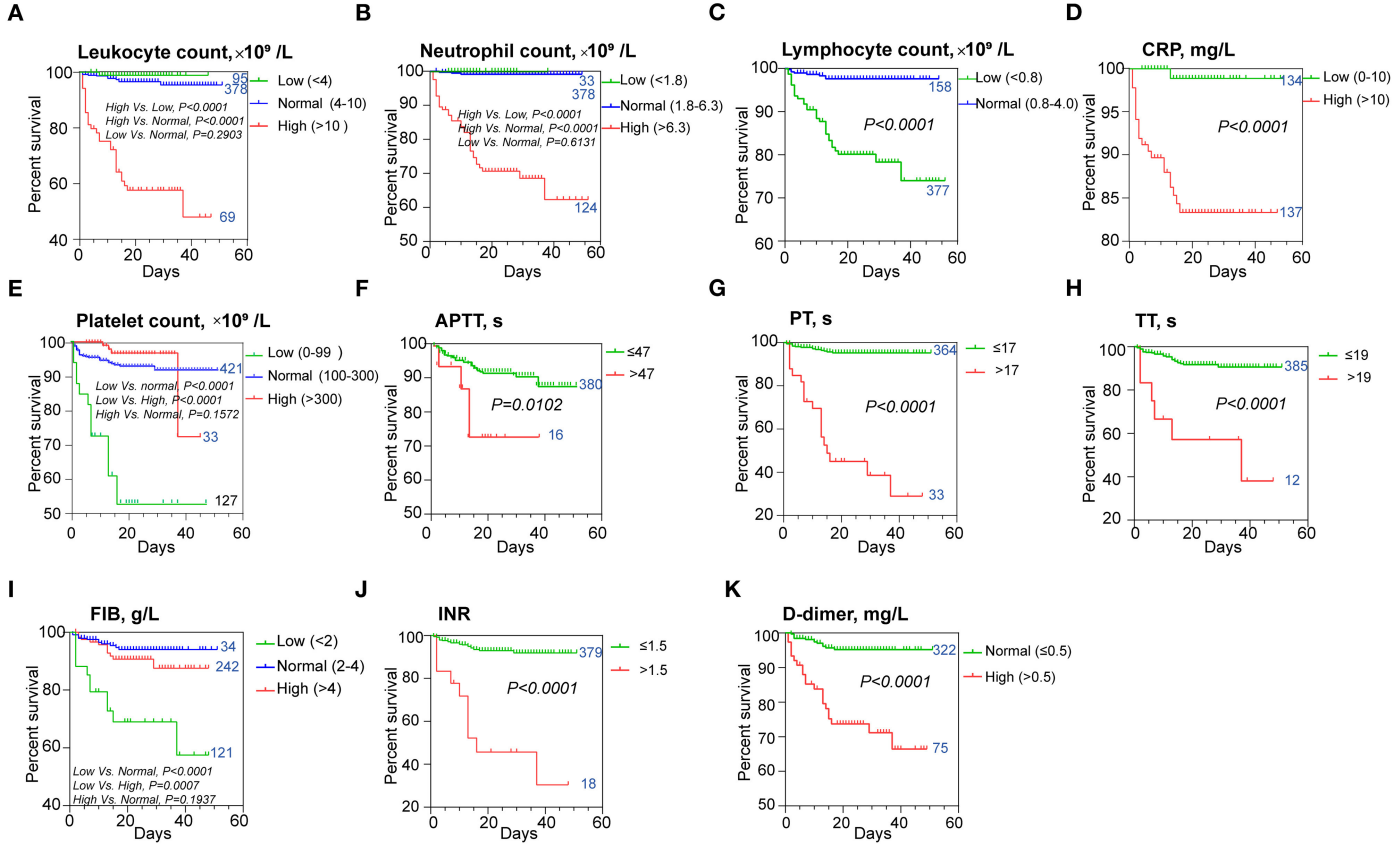
Kaplan–Meier survival curves for different prognostic factors. The curves according to **(A)** leukocytes, **(B)** neutrophils, **(C)** lymphocytes, **(D)** CRP levels, **(E)** platelets, **(F)** APTT, **(G)** PT, **(H)** TT, **(I)** FIB levels, **(J)** INR, and **(K)** D-dimer levels. The patient number of each group was indicated next to the curve. CRP, C-reactive protein; APTT, activated partial thromboplastin time; INR, international normalized ratio; PT, prothrombin time; TT, thrombin time; FIB, fibrinogen.

The Kaplan–Meier analysis with log-rank test for the hematological indicators showed a significant difference in survival curve in COVID-19 patients categorized by the levels of leukocyte (Figure 2A), neutrophil (Figure 2B), lymphocyte (Figure 2C), CRP (Figure 2D), and those indices related to coagulation function, including platelets (Figure 2E), APTT (Figure 2F), PT (Figure 2G), TT (Figure 2H), fibrinogen (FIB; Figure 2I), INR (Figure 2J), and D-dimer (Figure 2K), respectively. COVID-19 patients with a higher leukocyte count (>10 × 10^9^/l) had a worse prognosis. No significant difference in prognosis was observed in COVID-19 patients with lower leukocyte count (<4 × 10^9^/l) compared with those with normal leukocyte levels (Figure 2A). Similar results were found on neutrophil and FIB, that a low level of neutrophil count (<1.8 × 10^9^/l) and a high level of FIB (>4 g/l) did not significantly contribute to the worse prognosis than the normal-level group (Figures 2B,I). Our Kaplan–Meier analysis also showed that patients with high levels of CRP (>10 μg/l), D-dimer (>0.5 mg/l), extended APTT (>47 s), PT (>17 s), TT (>19 s), and high INR were associated with worse prognosis (Figures 2D,F–K). In contrast, normal levels of lymphocyte (>0.8 × 10^9^/l) and platelet (>100 × 10^9^/l) were associated with better prognosis (Figures 2A,E). Collectively, these results suggested that these hematological parameters and patients' characteristics could be a potential prognostic marker for COVID-19.

Furthermore, based on the multivariate Cox regression analysis, we found that among the 11 laboratory indices that could predict the prognosis of COVID-19 mentioned above, lymphocyte count <0.8 × 10^9^/l [hazard ratio (HR), 2.911; 95% confidence interval (CI), 1.172–7.229], neutrophil count >10 × 10^9^/l (HR, 15.679; 95% CI, 4.643–52.945), and PT > 17 s (HR, 6.864; 95% CI, 3.389–13.901) on admission were the risk factors for a fatal outcome (Table 2).

**Table 2:** Risk factors of fatal outcome in the multivariate cox proportional hazards regression model.

**Variables**	**Level**	**HR**	**95% CI**	***P*-value**
Lymphocyte count (×10^9^/l)	<0.8 vs. ≥0.8	2.911	1.172–7.229	0.021
Neutrophil count (×10^9^/l)	>6.3 vs. ≤ 6.3	15.679	4.643–52.945	<0.001
PT (s)	>17 vs. ≤ 17	6.864	3.389–13.901	<0.001

### Correlation of Characteristics and Hospitalization Days Within Discharged Patients

**Figure 3:**
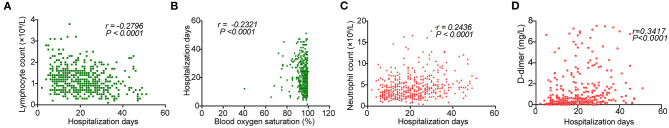
Correlations between hospitalization days and on admission levels of **(A)** leukocytes, **(B)** blood oxygen saturation, **(C)** neutrophils, and **(D)** D-dimers.

Here, we defined the correlations of characteristics and hospitalization days of the discharged patients by using Spearman's correlation analysis. Due to the limit of sample size, |*r*| > 0.2 and *P* < 0.05 were set as cutoff values for correlation. We observed that hospitalization days of discharged patients were negatively correlated with lymphocyte count and blood saturation levels on admission (*P* < 0.0001 and *P* = 0.0002, *r* < −0.2) (Figures 3A,B), while they were positively correlated with neutrophil count and D-dimer levels (*P* < 0.0001, *r* > 0.2; Figures 3C,D).

### Correlation Networks and Principal Component Analysis for Hematologic Indices

**Figure 4:**
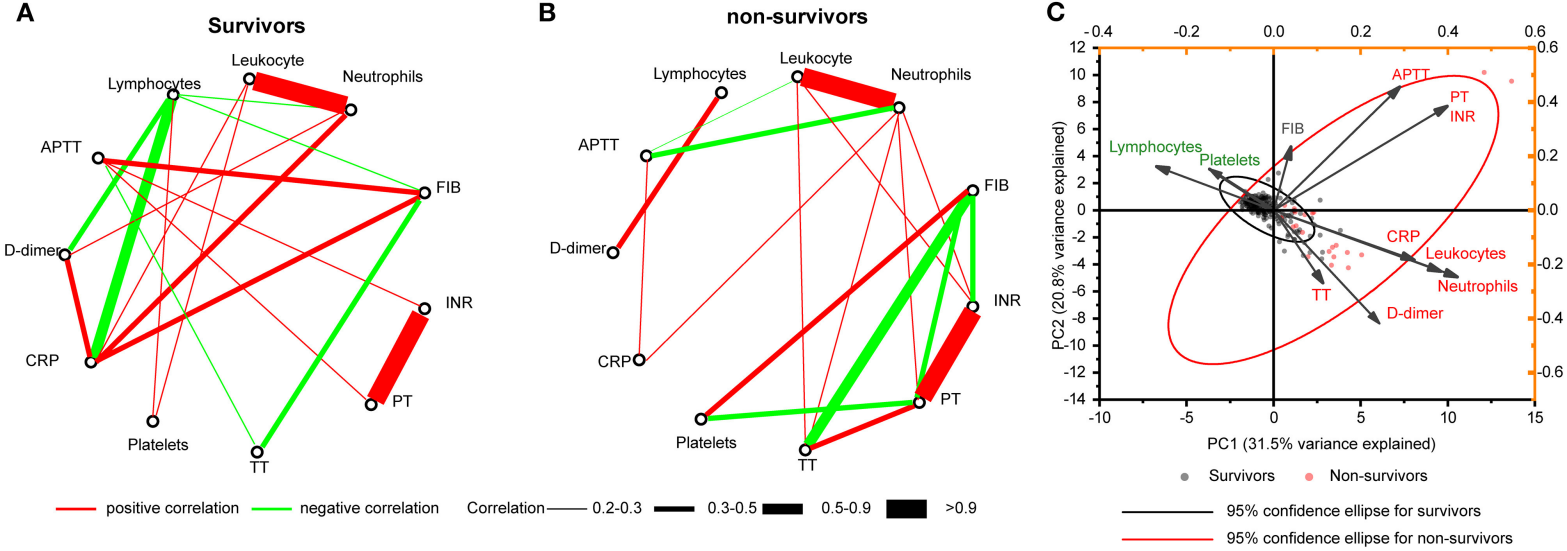
Correlation networks for hematologic indices. Networks showed different profiles of correlations in COVID-19 survivors **(A)** and non-survivors **(B)**, on admission. **(C)** PCA biplot of hematologic indices on admission. Individuals are shown as dots and colored by outcomes (survivors and non-survivors). Indices showed as lines with arrows and colored by positive or negative contribution to PC1. The configuration of indices in biplot represented the relationship between variables and principal components. PCA, principal component analysis.

Both survivors and non-survivors showed strong positive correlations between leukocytes and neutrophils (*r* = 0.94 and *r* = 0.99, respectively), and between INR and PT (both *r* = 1.00). Similarly, a moderate negative correlation between FIB and TT was found on both survivors and non-survivors (*r* = −0.31 and *r* = −0.59, respectively) (Figure 4).

We observed a negative correlation between lymphocytes and D-dimer in the survivors (*r* = −0.3338). However, this correlation was positive in the non-survivors (*r* = 0.4323). Lymphocyte counts and CRP levels had four and three more highly connected hub nodes in the survivors than in the non-survivors (six and five edges, respectively) (Figures 4A,B). In contrast, neutrophil counts and PT had two and three more connected hub nodes in the non-survivors (six and five edges, respectively) (Figures 4A,B). We did not observe a correlation between D-dimer and other coagulation indicators including platelets, APTT, TT, PT, INR, and FIB in both survivors and non-survivors (Figures 4A,B). We found that APTT lost correlation with other coagulation indicators including PT, TT, FIB, and INR in non-survivors (Figures 4A,B). A biplot via PCA indicated the configuration of hematologic indices on admission, which is shown in Figure 4C. The first principal component (PC1) could roughly separate non-survivors from survivors, with neutrophils (42.20%), leukocytes (38.42%), and CRP (32.32%) having the biggest positive contribution. In contrast, lymphocytes (26.91%) and platelets (14.71%) had a negative contribution to PC1.

### Cardiovascular-Related Underlying Comorbidities Were Associated With Poor Prognosis of COVID-19

**Figure 5:**
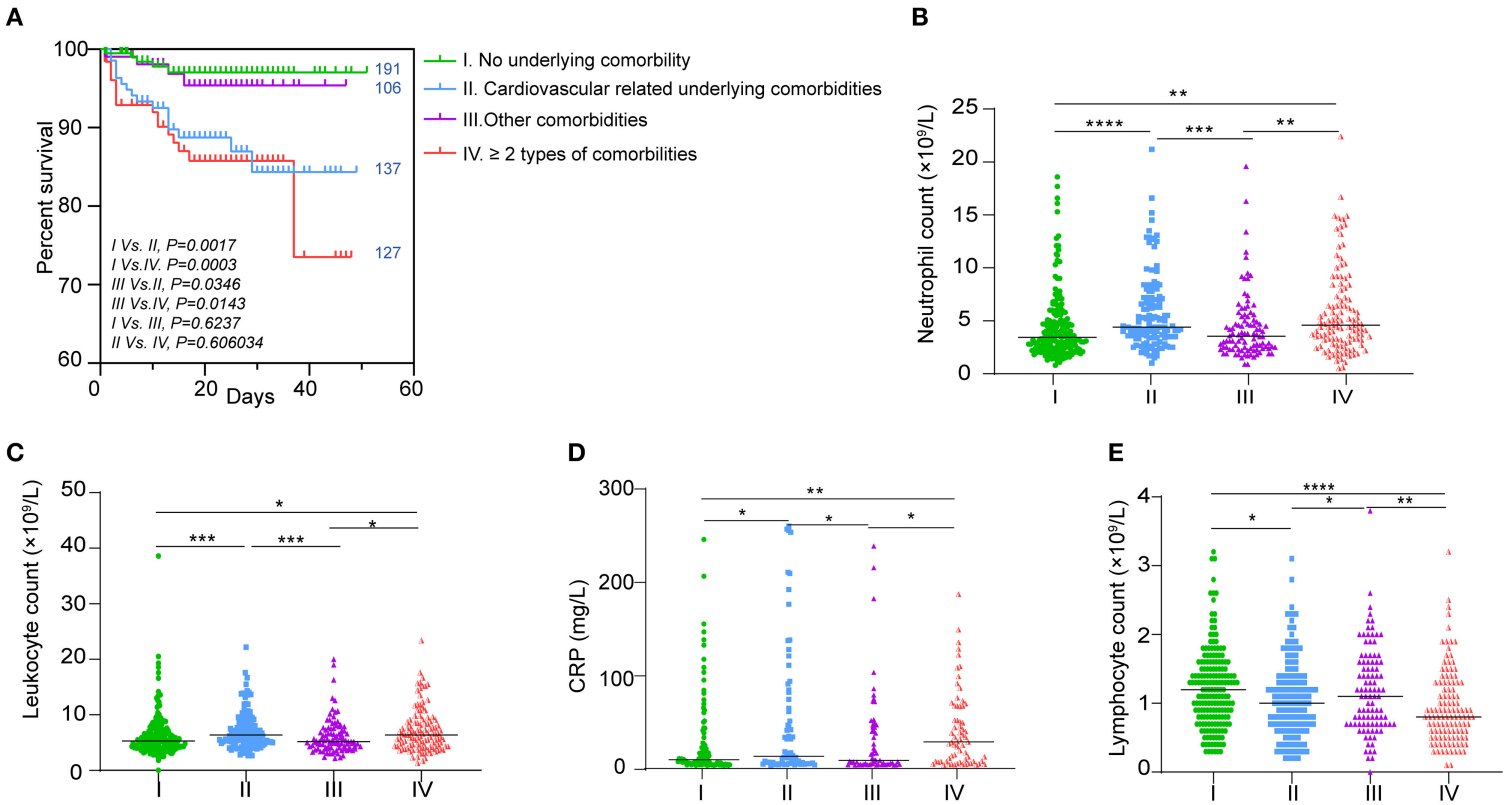
Association of underlying comorbidities and hematologic indices. **(A)** Kaplan–Meier survival curves for different underlying comorbidities. The number of patients in each group is indicated next to the curve. Hematologic indices in the four groups that showed to be better fit the survival curves including **(B)** neutrophils, **(C)** lymphocytes, **(D)** leukocytes, and **(E)** CRP levels. Data are shown as dots with median lines. *P < 0.05, **P < 0.01, ***P < 0.001, and ****P < 0.0001.

Based on the underlying comorbidity description, we categorized the patients into four groups including the no underlying comorbidity, CRUC, other-comorbidity group, more than two comorbidities, and at least one was CRUC. The Kaplan–Meier analysis with log-rank test showed a significantly different survival curve among the four groups (Figure 5). Except for the cardiovascular-related comorbidity (hypertension, diabetes, coronary heart disease, cerebrovascular disease, etc.), other comorbidities did not significantly affect the survival rate of the patients as compared to those without. Although a relatively low survival rate was observed in patients with more than two types of comorbidities as compared to those without, it had no significant difference as compared with those with only CRUC (Figure 5A). These results together indicated that CRUC might be the main factor that decreased the survival rate of COVID-19 patients.

To explore why the four groups of patients had such significantly different outcomes, we analyzed the hematologic indices that were associated with mortality in these patients. As shown in Supplementary Figure 1 and Supplementary Table 1, we did not find a significant difference in coagulation indices such as platelet count, PT, TT, D-dimer, INR, and FIB between patients without any comorbidity and those only with CRUC. Combined with the above results, the role of coagulation dysfunction in decreasing the survival rate of patients with CRUC was excluded.

In contrast, we found that neutrophil, lymphocyte, leukocyte, and CRP levels in the four groups all fit the trend of survival curves better (Figures 5B–D). Significantly high levels of neutrophil, leukocyte, and CRP were found in patients with CRUC as compared with those without any comorbidity. Comparable levels of neutrophil, leukocyte, and CRP were found in patients with an underlying comorbidity other than CRUC as compared with those without any comorbidity (Figures 5B–D). Similarly, we also found comparable levels of neutrophil, leukocyte, and CRP between patients with more than two types of comorbidities and those with only CRUC. Lymphocyte levels in the four groups showed the opposite trend (Figure 5E).

### The Age-Related Poor Prognosis of COVID-19 Patients Was Associated With CRUC

**Figure 6:**
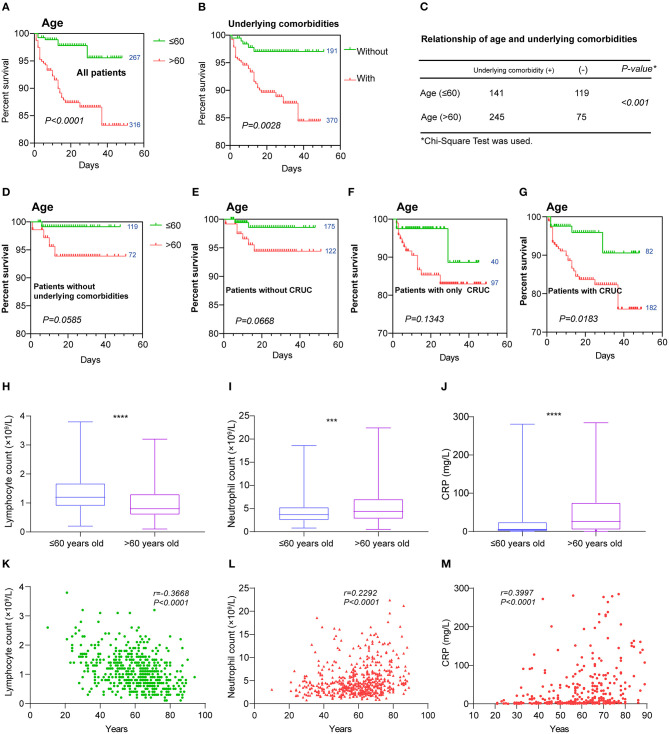
Hematologic variations between the young and old. Kaplan–Meier survival curves for **(A)** age and **(B)** underlying comorbidities are shown. **(C)** The relationship of age and underlying comorbidities was analyzed by chi-square test. Kaplan–Meier survival curves for age in those patients **(D)** without any underlying comorbidities, **(E)** those without cardiovascular-related comorbidities, **(F)** those with only cardiovascular-related underlying comorbidities, and **(G)** those with cardiovascular-related underlying comorbidities. Hematologic indices that were found to have different levels between the young and old including **(H)** lymphocytes, **(I)** neutrophils, and **(J)** CRP and were also correlated with age **(K–M)**. Data are shown as boxes and whiskers. Correlations are colored by positive (red) or negative (green). The numbers next to the survival curves indicate quantities of patients in such a group. ***P < 0.001 and ****P < 0.0001.

The Kaplan–Meier analysis with log-rank tests showed that elderly patients (>60 years of age) had a poorer outcome than those who are younger (≤60) (Figure 6A), that at day 47 after admission, 95.58% of young patients survived, while only 83.24% of elderly patients did. We also found significantly different survival curves between patients with or without underlying comorbidities (Figure 6B). We hypothesized that age-related poor prognosis might be related to a higher frequency of underlying comorbidities that happened in the older age group, which was observed in our dataset (Figure 6C). To validate this hypothesis, we analyzed the survival curves of the young and the old with each category of underlying comorbidities. We found no significant difference in survival curves between the young and the old who had no underlying comorbidities (Figure 6D). Similar results were also observed in the patients without CRUC and patients with only CRUC (Figures 6E,F). However, we found a significant difference in survival curves between the young and the old who had CRUC (Figure 6G). Altogether, these results highlighted the contribution of CRUC in the age-related poor prognosis. Then, we analyzed the hematologic index differences between the old and the young. We found a significantly lower level of lymphocyte and a higher level of neutrophil and CRP in the old (Figures 6H–J). Besides, significant correlations (*P* < 0.05, |*r*| > 0.2) between the age and these hematologic indices (lymphocyte count, neutrophil count, and CRP level) were found in the patients (Figures 6K–M). However, most other hematologic indices were found not to be significantly different between the young and old or have a correlation with age (Supplementary Figures 2A–O, Supplementary Table 2), except the D-dimer level (Supplementary Figure 2P).

### Male COVID-19 Patients Had a Poorer Outcome

**Figure 7:**
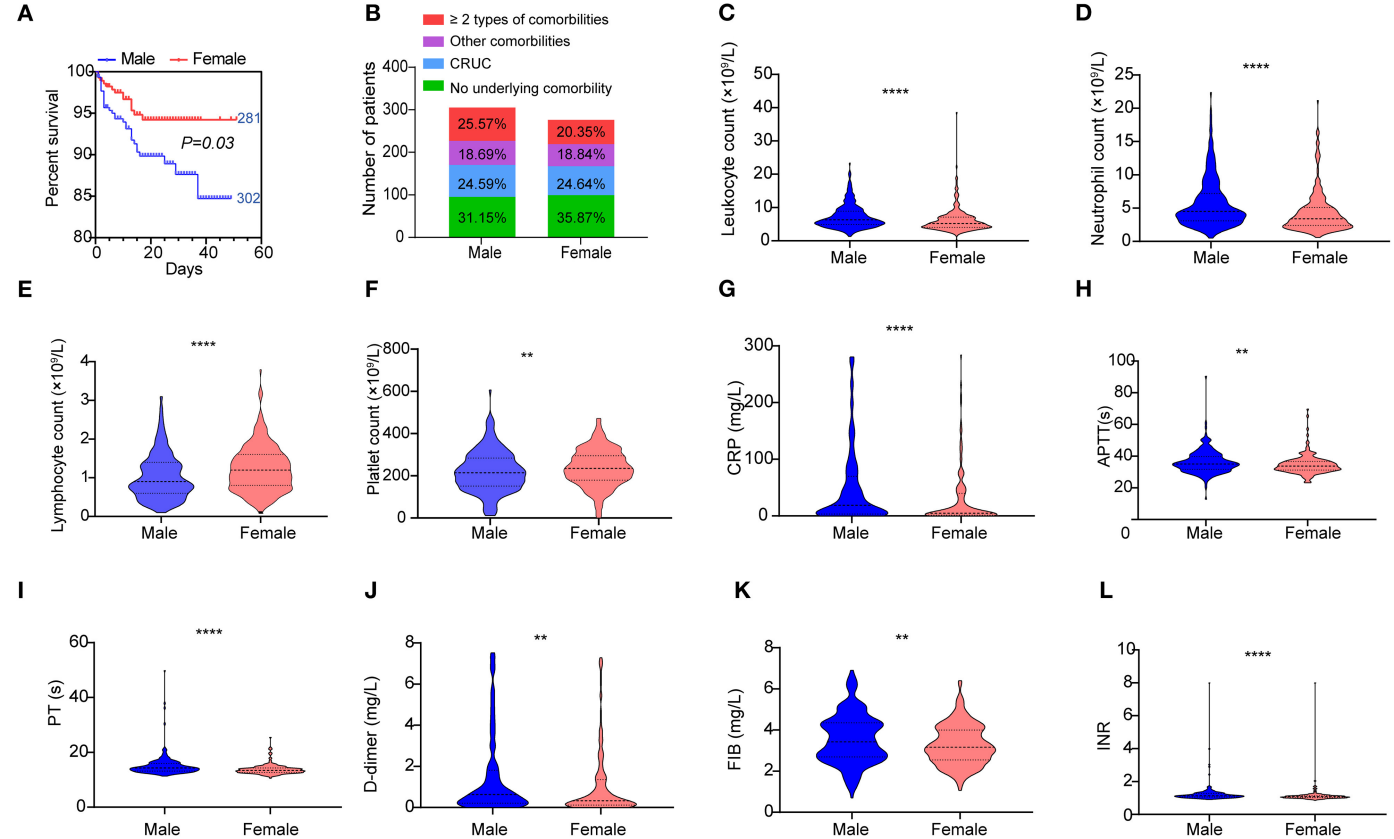
Hematologic variations between the male and female. **(A)** Kaplan–Meier survival curves for genders are shown. **(B)** Composition of different underlying comorbidities in the male and female. Different levels of hematologic indices **(C)** leukocytes, **(D)** neutrophils, **(E)** lymphocytes, **(F)** platelets, **(G)** CRP level, **(H)** APTT, **(I)** PT, **(J)** D-dimer level, **(K)** FIB, and **(L)** INR between the male and female. Data are shown as a violin plot with median and 25 and 75% percentile lines. **P < 0.01, ****P < 0.0001. The numbers next to the survival curves indicate quantities of patients in such a group.

As shown in Figure 7A, a significant difference in survival curves was observed between male and female patients, suggesting that male patients had a poorer outcome than female. We did not observe different occurrences of underlying comorbidities between the male and female (Figure 7B, Supplementary Table 3). To explore a possible explanation, we analyzed hematologic indices between the male and female. By using the Mann–Whitney *U*-test, we found that male patients had significantly higher levels of leukocytes, neutrophils, CRP, D-dimer, FIB, and INR; extended APTT and PT; and lower levels of platelets and lymphocytes (Figures 7C–L). However, when counting the frequencies of normal and abnormal levels of these indices, we found that most coagulation indices including platelet, APTT, TT, and INR were not significantly different between the male and female (Supplementary Table 3).

Taken together, these results indicated that higher inflammatory conditions that manifested as higher levels of leukocytes, neutrophils, CRP, and D-dimer and lower lymphocyte count were the main factors associated with the poorer outcome of male patients. In contrast, coagulation disorders might have a limited contribution to the poorer outcome of male patients.

## Discussion

In this study, hematologic biomarkers associated with the progression of COVID-19 were investigated, and some novel findings were documented. First, patient characteristics including the hematologic indices that could predict the fatal outcome of COVID-19 or be associated with the patient's duration of hospitalization in discharged people were detailed and documented in the present study. Second, lymphopenia, hyper inflammation status, and coagulation derangements were shown to be associated with fatal outcome of COVID-19 patients, and their contribution to the fatal outcome of different types of patients (patients with different types of underlying comorbidities, young or old, and male or female) was elucidated.

In our study, results were also found with the incidence of 30.73% (lymphopenia), 24.18% (neutrophilia) (17), and 6.19% (thrombocytopenia) (18) among all the patients, respectively. Higher incidences of these hematological changes were found in deceased patients as compared to the discharged patients (Table 1). A higher neutrophil level on admission was found in deceased patients and could predict poor outcomes in our cohort (Figure 2B). The involvement of elements of the hematopoietic system is prominent in severe cases and associated with poor outcomes and mortality (19). Blood counts and coagulation parameters are also frequently dysregulated in severe COVID-19 (20, 21). A severe disease is commonly complicated by lymphopenia (22), thrombocytopenia, and coagulopathy, often progressing to disseminated intravascular coagulation (DIC) (23).

Our study also indicated that decreased platelet count might be able to serve as a potential clinical indicator of mortality during hospitalization (Figure 2E). This result was also consistent with our previous studies (15, 16). The mechanism of the reduction of platelet counts in COVID-19 patient may include (1) the inhibition of hematopoiesis in the bone marrow through certain receptors causes decreased primary platelet formation (24, 25); (2) the hyperreactivity of platelets increases the consumption of platelets/megakaryocytes; and (3) the lung functions as one of the hematopoietic organs (26), and SARS-CoV-2 may disrupt its function like SARS. An abnormal coagulation status is an important phase for COVID-19 patients (27). Many coagulation indices, including APTT, PT, TT, FIB, and INR, and some other hematologic indices, including leukocyte, CRP, and D-dimer on admission, were shown to be different between the survivors and non-survivors and could be used as prognostic indicators for a fatal outcome of COVID-19 (Table 1, Figure 1). However, the multivariate Cox regression model suggested that lymphopenia, neutrophilia, and prolonged PT serve as the predictors of fatal outcome (Table 2). This was partially consistent with the results obtained by the correlation networks and PCA, that these three indices had more connected hub nodes in the survivors or non-survivors or had the biggest positive/negative contribution for PC1 (Figure 4). Furthermore, findings on the correlation of hematologic characteristics and hospitalization days confirmed the role of these biomarkers for predicting the prognosis and might help us to build a model to predict the length of hospitalization (Figures 2, 3).

Patients with underlying comorbidities may have a worse outcome than those without (28, 29). Our present study provides further evidence to substantiate this notion (Table 1, Figure 5A). We observed that CRUC, but not other comorbidities, might contribute to higher mortality for COVID-19 patients. We did not categorize the comorbidities into smaller types, but into four major categories mentioned above (Table 1). Then, we researched the survival curves of the patients with each category of comorbidity. It was shown that only CRUC, but not other comorbidities, was associated with poorer clinical outcomes (Figure 5A).

Hematological indicators fitted this result perfectly including lymphocyte, leukocyte, and neutrophil counts and CRP levels (Figures 5B–E). These indicators were found to have identical trends among the survival curves of the four comorbidity categories. Thus, these four comorbidity clinical indicators might carry the implications of specific hematological changes and their associated poor outcomes in patients with such comorbidities. First, the decrease in lymphocyte, especially in the T cells, might be frequently found in those patients with CRUC (30, 31). These might represent the defects of these cells, which might in turn cause T cells to unable to efficiently combat viral infections (31). Second, patients with CRUC also showed higher inflammation levels in our study, which was manifested as elevated neutrophil count and CRP levels (Figures 5D,E). Inflammatory processes and systematic inflammation play a central role in CRUC (32, 33). Hyperinflammation that drives lung or multiorgan injury was often found on COVID-19 patients with worse outcomes (34). Therefore, we could conclude that CRUC contributing to worse outcomes might be related to lymphocyte dysfunction and high background inflammatory state.

Our results confirmed that older age was associated with increased death (Figure 6A). This may be associated with age-related underlying comorbidities, particularly the CRUC (Figures 6D–G) and age-dependent defects in T and B-cell function (Figures 6H,K). As markers of inflammatory reactions, neutrophil and CRP levels were higher in deceased patients and associated with fatal outcome (Figures 1B,E, 2B,D, Table 1). They were found to be positively correlated with COVID-19 patients' age (*r* = 0.2292 and 0.3997, *P* < 0.0001) in our study (Figures 6L,M). This result further confirmed that irresistible and overexuberant inflammatory response was a potential risk factor that caused the death in SARS-CoV-2 infection given that viral load might not be correlated with the worsening of symptoms, which highlighted the rationality of combining antiviral and anti-inflammatory treatments for COVID-19 (35, 36). Another evidence of a higher inflammatory status of the old was found in the D-dimer level, which was higher and positively correlated with age (Supplementary Figure 2P). In COVID-19 patients, D-dimer was found to be related to markers of inflammation (37, 38). Thus, strengthening cellular immunity and anti-inflammation could be an option for COVID-19 therapy, especially for the old with CRUC (39). In contrast, coagulation disorders were shown not to be the main factors that contributed to the different outcomes between the old and young (Supplementary Figure 2). Furthermore, the reverse correlation of lymphocyte and D-dimer between the survivors and non-survivors was interesting, and we proposed that it was caused by an incongruent decrease of lymphocyte and an increase of D-dimer happened in non-survivors.

We also found that male patients had a worse outcome than female or the young (Figure 7A). No association of underlying comorbidities and gender was found in the cohort, indicating underlying comorbidities may not contribute to such difference. An explanation showed that the female patients mounted significantly more robust T-cell activation than male patients during SARS-CoV-2 infection, which was sustained in the old (40). As we knew, a large proportion (>70%) of lymphocytes were T-cells. We also found that lymphocyte level in female COVID-19 patients was higher than that in the male. However, this was not observed in the deceased patient (Supplementary Figure 3). Taken together, we speculated that the lymphocyte level of COVID-19 patients might reflect the level of activated T-cells targeting virus-infected cells (41). Additionally, we also found levels of many hematologic indices to be different between the male and female, which indicated that they might contribute to different outcomes (42).

However, the main factors that contributed to the worse outcome of male patients were lymphocyte dysfunction and hyperinflammation, while coagulation disorders might have partly contributed as most of the coagulation indices were not significantly different between the male and female. The finding of higher inflammatory conditions in the male than in the female patients may be associated with sex hormone differences. Differing in their immunological reactions to foreign and self-antigens, males and females are distinctively different in innate and adaptive immune responses. Importantly, these sex-based immunological differences contribute to variations in the incidence of autoimmune diseases and malignancies, susceptibility to infectious diseases, and responses to vaccines in males and females (43). Besides, X-chromosome mosaicism in the female is associated with varied genes involved in inflammation. This biased response from X chromosome also promotes differential immunological responses observed in women and men (44). Taken together, these results might indicate different treatment strategies for different types of patients. For example, for patients with CRUC, immune-supportive treatment and anti-inflammatory therapy were of ultimate importance, while for the old and male patients, besides the two above treatment strategies, coagulation support treatment could not be ignored.

## Data Availability Statement

The original contributions presented in the study are included in the article/Supplementary Material, further inquiries can be directed to the corresponding author/s.

## Ethics Statement

The studies involving human participants were reviewed and approved by the Ethics Committee of the Seventh Affiliated Hospital, Sun Yat-sen University. The patients/participants provided their written informed consent to participate in this study.

## Author Contributions

X-YZ, LL, BH, YHe, and MY had the idea and designed the study. X-YZ, LL, and HK contributed to the writing of the manuscript. BH, MN, MY, CC, YHe, YHu, and QL contributed to the critical revision of the manuscript. X-YZ and LL contributed to the statistical analysis. X-YZ, BH, LL, and MY have verified the underlying data. All authors contributed to the article and approved the submitted version.

## Conflict of Interest

The authors declare that the research was conducted in the absence of any commercial or financial relationships that could be construed as a potential conflict of interest.

## Supplementary Material

Supplementary Figure 1 Hematologic indices in four categories of underlying comorbidities that showed to not fit the survival curves including **(A)** platelets, **(B)** APTT, **(C)** PT, **(D)** TT, **(E)** D-dimer, **(F)** FIB, and **(G)** INR. Data are shown as dots with median lines. ^**^*P* < 0.01, ^****^*P* < 0.0001.

Supplementary Figure 2 Hematologic indices that were shown to be not different between the young and old or the levels of which were not significantly correlated with age (*P* > 0.05 or |*r*| < 0.2), including **(A,I)** leukocytes, **(B,J)** platelets, **(C,K)** APTT, **(D,L)** PT, **(E,M)** TT, **(F,N)** FIB, **(G,O)** INR. **(H,P)** D-dimer levels between the young and old and its correlation with age. Data are shown as boxes and whiskers. Correlations are colored by positive (red) or negative (green) or no correlation (*P* > 0.05) (gray). ^*^*P* < 0.05, ^****^*P* < 0.0001.

Supplementary Figure 3 Lymphocyte levels in the male and female survivors and non-survivors. ^****^*P* < 0.0001; n.s., not significant.

Supplementary Table 1 Baseline characteristics of patients with or without cardiovascular-related underlying comorbidities (CRUC). ^#^COVID-19 mortality of patients with high neutrophil counts (>6.3 × 10^9^/l) or leukocyte counts (>10 × 10^9^/l) was compared with other two groups. ^$^COVID-19 mortality of low-platelet-count group (<100 × 10^9^/l) and low FIB (<2 g/l) was compared with the other two groups. ^∧^COVID-19 mortality of high-platelet-count group (>300 × 10^9^/l) and low FIB (>4 g/l) was compared with the other two groups.

Supplementary Table 2 Baseline characteristics of old and young patients. ^*^Fisher's exact test was used to compare the COVID-19 mortality between the patients with different indices. ^&^Patients with or without comorbidities were compared. ^@^Patients with or without CRUC were compared. ^#^COVID-19 mortality of patients with high neutrophil counts (>6.3 × 10^9^/l) or leukocyte counts (>10 × 10^9^/l) was compared with the other two groups. ^$^COVID-19 mortality of low-platelet-count group (<100 × 10^9^/l) and low FIB (<2 g/l) was compared with the other two groups. ^∧^COVID-19 mortality of high-platelet-count group (>300 × 10^9^/l) and low FIB (>4 g/l) was compared with the other two groups.

Supplementary Table 3 Baseline characteristics of male and female patients. ^*^Chi-square tests or Fisher's exact test were used to compare the COVID-19 mortality between the patients with different indices. ^&^Patients with or without underlying comorbidities were compared. ^@^Patients with or without CRUC were compared. ^#^COVID-19 mortality of patients with high neutrophil counts (>6.3 × 10^9^/l) or leukocyte counts (>10 × 10^9^/l) was compared with the other two groups. ^$^COVID-19 mortality of low-platelet-count group (<100 × 10^9^/l) and low FIB (<2 g/l) was compared with the other two groups. ^∧^COVID-19 mortality of high-platelet-count group (>300 × 10^9^/l) and low FIB (>4 g/l) was compared with the other two groups.
