# Supplementary figures and images for: Elderly Male With Cardiovascular-Related Comorbidities Has a Higher Rate of Fatal Outcomes: A Retrospective Study in 602 Patients With Coronavirus Disease 2019
Source: Front Cardiovasc Med. 2021 Jun 7;8:680604. doi: 10.3389/fcvm.2021.680604 (PMC8215131; doi:10.3389/fcvm.2021.680604)

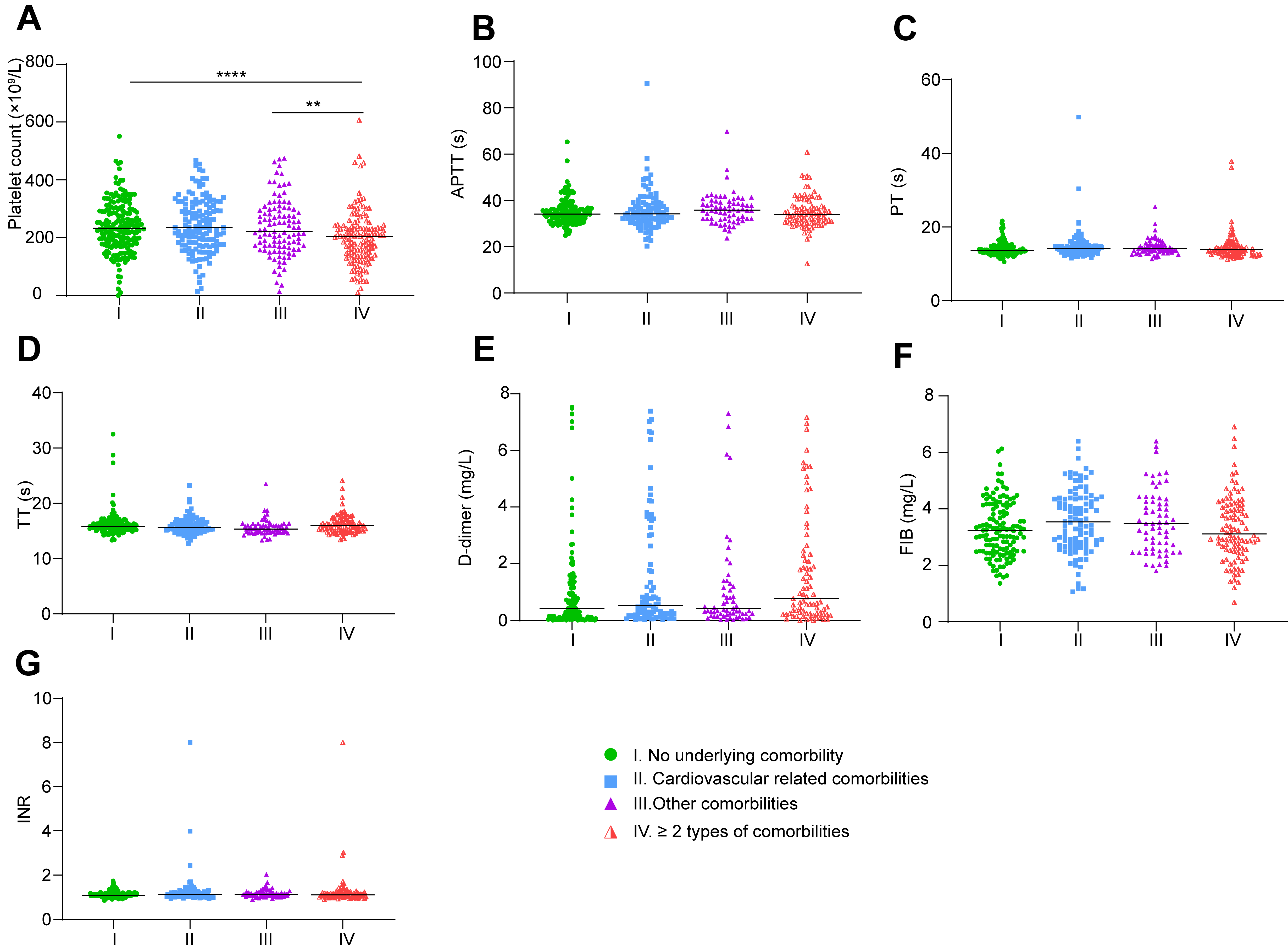

Supplement: Supplementary Figure 1 — Hematologic indices in four categories of underlying comorbidities that showed to not fit the survival curves including (A) platelets, (B) APTT, (C) PT, (D) TT, (E) D-dimer, (F) FIB, and (G) INR. Data are shown as dots with median lines. **P < 0.01, ****P < 0.0001. [file Image_1.TIF]

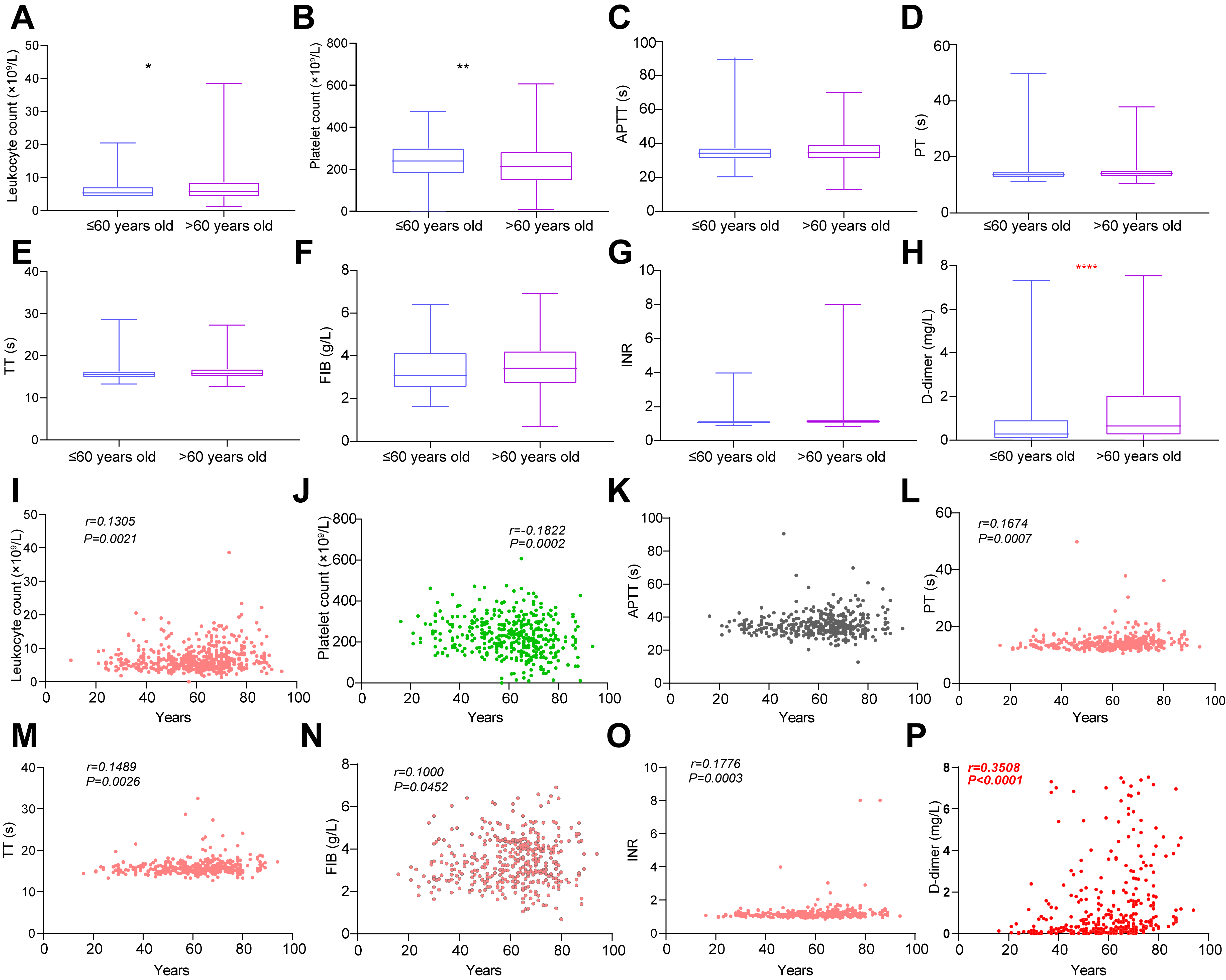

Supplement: Supplementary Figure 2 — Hematologic indices that were shown to be not different between the young and old or the levels of which were not significantly correlated with age (P > 0.05 or |r| < 0.2), including (A,I) leukocytes, (B,J) platelets, (C,K) APTT, (D,L) PT, (E,M) TT, (F,N) FIB, (G,O) INR. (H,P) D-dimer levels between the young and old and its correlation with age. Data are shown as boxes and whiskers. Correlations are colored by positive (red) or negative (green) or no correlation (P > 0.05) (gray). *P < 0.05, ****P < 0.0001. [file Image_2.TIF]

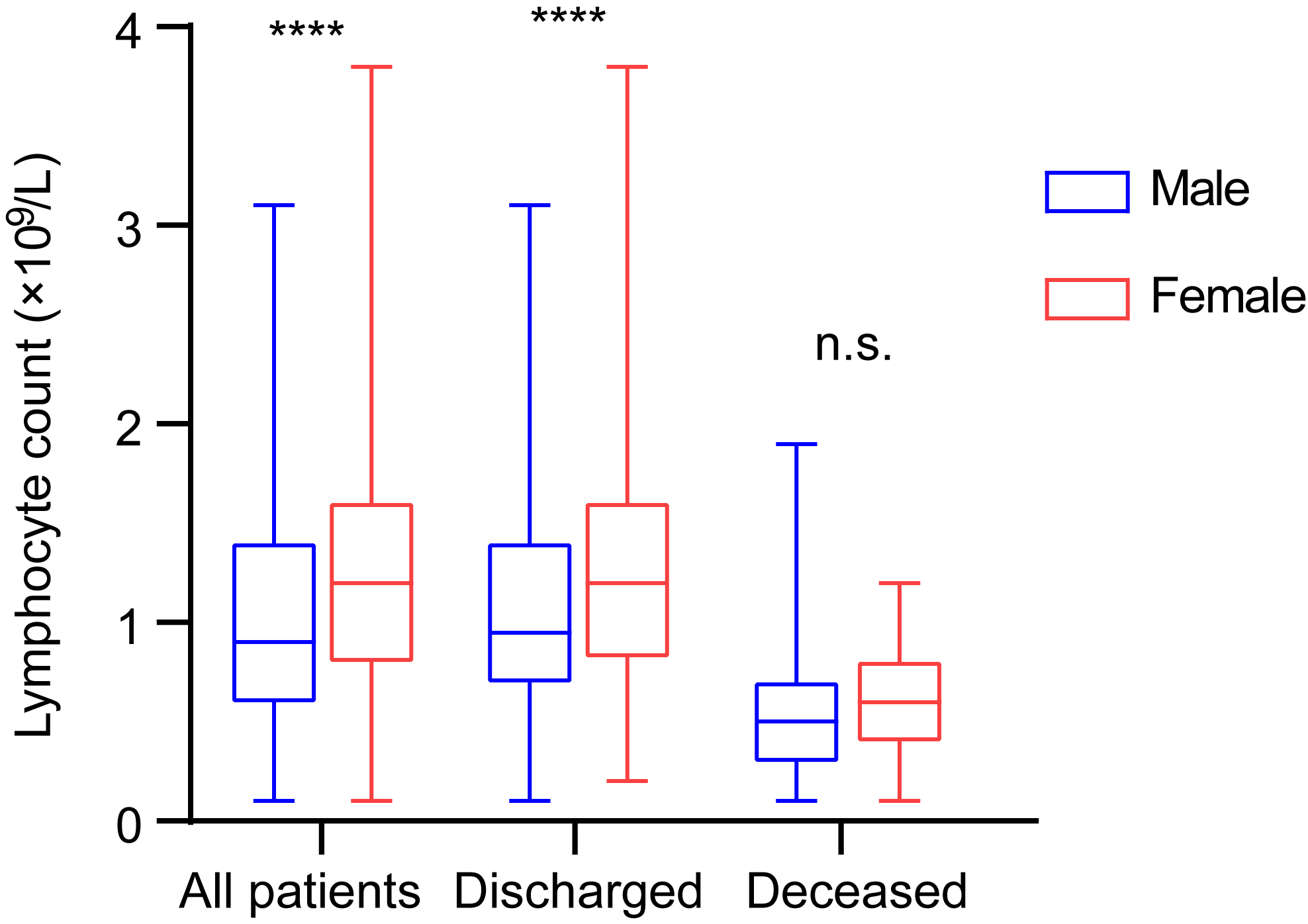

Supplement: Supplementary Figure 3 — Lymphocyte levels in the male and female survivors and non-survivors. ****P < 0.0001; n.s., not significant. [file Image_3.TIF]
