# Supplementary material for: Elderly Male With Cardiovascular-Related Comorbidities Has a Higher Rate of Fatal Outcomes: A Retrospective Study in 602 Patients With Coronavirus Disease 2019
Source: Front Cardiovasc Med. 2021 Jun 7;8:680604. doi: 10.3389/fcvm.2021.680604 (PMC8215131; doi:10.3389/fcvm.2021.680604)
Supplement: Supplementary Table 1 — Baseline characteristics of patients with or without cardiovascular-related underlying comorbidities (CRUC). #COVID-19 mortality of patients with high neutrophil counts (>6.3 × 10^9^/l) or leukocyte counts (>10 × 109/l) was compared with other two groups. $COVID-19 mortality of low-platelet-count group (<100 × 109/l) and low FIB (<2 g/l) was compared with the other two groups. ∧COVID-19 mortality of high-platelet-count group (>300 × 109/l) and low FIB (>4 g/l) was compared with the other two groups. [file Table_1.DOCX]

**Table S1. Baseline characteristics of patients with or without cardvascular related underlying comorbidities (CRUC).**

| Characteristics | All patients (n=602) | No CRC (n=302) | CRC (n=278) | *P*-value |
| --- | --- | --- | --- | --- |
| **Demographic** |  |  |  |  |
| **Fatal outcome** |  |  |  |  |
| Discharged, n (%) | 519 (89.48) | 288 (95.36) | 231 (83.09) | <0.001 |
| Deceased, n (%) | 61 (10.52) | 14 (4.64) | 47 (16.91) |  |
| **Age** |  |  |  |  |
| ≤ 60, n(%) | 260 (44.83) | 176 (58.28) | 84 (30.22) | <0.001 |
| >60, n(%) | 320 (55.17) | 126 (41.72) | 194 (69.78) |  |
| **Laboratory findings** |  |  |  |  |
| **Hematologic** |  |  |  |  |
| **Leukocyte count, 10⁹ /L** |  |  |  |  |
| <4, n (%) | 95 (17.92) | 62 (21.68) | 33 (13.52) | 0.006# |
| 4 -10, (%) | 365 (68.87) | 197 (68.88) | 168 (68.85) |  |
| >10, n (%) | 70 (13.21) | 27 (9.44) | 43 (17.62) |  |
| **Neutrophil count, ×10⁹/L** |  |  |  |  |
| <1.8, n (%) | 33 (6.25) | 20 (7.04) | 13 (5.33) | <0.001# |
| 1.8-6.3, (%) | 368 (69.70) | 214 (75.35) | 154 (63.11) |  |
| >6.3, n (%) | 127 (24.05) | 50 (17.61) | 77 (31.56) |  |
| **Lymphocyte count, ×10⁹ /L** |  |  |  |  |
| <0.8, n (%) | 160 (30.30) | 71 (25.00) | 89 (36.48) | 0.004 |
| 0.8-4.0 (%) | 368 (69.70) | 213 (75.00) | 155 (63.52) |  |
| **Platelet count, ×10⁹ /L** |  |  |  |  |
| <100, n (%) | 33 (5.77) | 13 (4.35) | 21 (7.66) | **0.093$, 0.365^** |
| 100-300, n (%) | 419 (73.25) | 220 (73.58) | 199 (72.63) |  |
| >300, n (%) | 120 (20.98) | 66 (22.07) | 54 (19.71) |  |
| **Other indices** |  |  |  |  |
| APTT, s |  |  |  |  |
| ≤47, n (%)  47s, n (%) | 371 (94.64) | 194 (97.00) | 177 (92.19) | 0.034 |
| >47, n (%) | 21 (5.36) | 6 (3.00) | 15 (7.81) |  |
| **Prothrombin time (PT), s** |  |  |  |  |
| ≤17, n (%) | 355 (94.64) | 185 (92.50) | 170 (88.54) | **0.243** |
| >17, n (%) | 37 (5.36) | 15 (7.50) | 22 (11.46) |  |
| **Trombin time (TT), s** |  |  |  |  |
| ≤19, n (%) | 379 (96.44) | 194 (96.52) | 185 (96.35) | **1.000** |
| >19, n (%) | 14 (3.56) | 7 (3.48) | 7 (3.65) |  |
| **D-dimer, mg/L** |  |  |  |  |
| <0.5, n (%) | 160 (41.88) | 99 (49.50) | 71 (36.98) | 0.012 |
| ≥0.5, n (%)  0.5mg/L, n(%) | 222 (58.12) | 101 (50.50) | 121 (63.02) |  |
| **Fibrinogen (FIB), g/L** |  |  |  |  |
| <2, n (%) | 35 (9.02) | 17 (8.50) | 18 (9.57) | **0.712$, 0.198^** |
| 2-4, n (%) | 231 (59.54) | 126 (63.00) | 105 (55.85) |  |
| >4, n (%) | 122 (31.44) | 57 (28.20) | 65 (34.57) |  |
| **International normalized ratio (INR)** |  |  |  |  |
| ≤1.5, n (%) | 370 (94.39) | 191 (95.50) | 179 (93.23) | **0.329** |
| >1.5, n (%) | 22 (5.61) | 9 (4.50) | 13 (6.77) |  |
| **C-reactive protein, mg/L** |  |  |  |  |
| ≤10, n (%)  , n (%) | 143 (48.64) | 86 (54.78) | 57 (41.61) | 0.024 |
| >10, n (%) | 151 (51.36) | 71 (45.22) | 80 (58.39) |  |

# COVID-19 mortality of patients with high neutrophil counts (>6.3×10⁹/L) or leukocyte counts（>10×10⁹/L) were compared with other two groups.

$ COVID-19 mortality of low platelet count group(<100×10⁹/L) and low FIB (<2g/L) were compared with the other two groups.

^ COVID-19 mortality of high platelet count group(>300×10⁹/L) and low FIB (>4g/L) were compared with the other two groups.
