# Supplementary material for: Elderly Male With Cardiovascular-Related Comorbidities Has a Higher Rate of Fatal Outcomes: A Retrospective Study in 602 Patients With Coronavirus Disease 2019
Source: Front Cardiovasc Med. 2021 Jun 7;8:680604. doi: 10.3389/fcvm.2021.680604 (PMC8215131; doi:10.3389/fcvm.2021.680604)
Supplement: Supplementary Table 2 — Baseline characteristics of old and young patients. *Fisher's exact test was used to compare the COVID-19 mortality between the patients with different indices. &Patients with or without comorbidities were compared. @Patients with or without CRUC were compared. #COVID-19 mortality of patients with high neutrophil counts (>6.3 × 10^9^/l) or leukocyte counts (>10 × 109/l) was compared with the other two groups. $COVID-19 mortality of low-platelet-count group (<100 × 109/l) and low FIB (<2 g/l) was compared with the other two groups. ∧COVID-19 mortality of high-platelet-count group (>300 × 109/l) and low FIB (>4 g/l) was compared with the other two groups. [file Table_2.DOCX]

**Table S2. Baseline characteristics of old and young patients**

| Characteristics | All patients (n=602) | Young (n=270) | Old (n=332) | *P*-value |
| --- | --- | --- | --- | --- |
| **Demographic** |  |  |  |  |
| **Comorbidity** |  |  |  |  |
| No comorbidities, n (%) | 194 (33.44) | 119 (45.77) | 75 (23.44) | <0.001@, <0.001&, |
| CRUC, n (%) | 143 (24.66) | 41 (15.77) | 102 (31.88) |  |
| Other comorbidities, n (%) | 108 (18.62) | 57(21.92) | 51 (15.94) |  |
| Two and more comorbidities, n (%) | 135 (23.28) | 43 (16.54) | 92 (28.75) |  |
| **Laboratory findings** |  |  |  |  |
| **Hematologic** |  |  |  |  |
| **Leukocyte count, 10⁹ /L** |  |  |  |  |
| <4, n (%) | 95 (21.2) | 47 (18.29) | 48 (16.27) | 0.001# |
| 4 -10, (%) | 284 (62.83) | 189 (73.54) | 195 (66.10) |  |
| >10, n (%) | 73 (16.15) | 21 (8.17) | 52 (17.63) |  |
| **Neutrophil count, ×10⁹/L** |  |  |  |  |
| <1.8, n (%) | 33 (6.00) | 19 (7.42) | 14 (4.76) | <0.001# |
| 1.8-6.3, (%) | 384 (69.82) | 193 (75.39) | 191 (64.97) |  |
| >6.3, n (%) | 133 (24.18) | 44 (17.19) | 89 (30.27) |  |
| **Lymphocyte count, ×10⁹ /L** |  |  |  |  |
| <0.8, n (%) | 169 (30.73) | 46 (17.97) | 123 (41.84) | <0.001 |
| 0.8-4.0 (%) | 381 (69.27) | 210 (82.03) | 171 (58.16) |  |
| **Platelet count, ×10⁹ /L** |  |  |  |  |
| <100, n (%) | 33 (5.56) | 8 (2.99) | 26 (7.95) | 0.012$, **0.075^** |
| 100-300, n (%) | 432 (72.73) | 193 (72.01) | 239 (73.09) |  |
| >300, n (%) | 129 (21.72) | 67 (25.00) | 62 (18.96) |  |
| **Other indices** |  |  |  |  |
| APTT, s |  |  |  |  |
| ≤47, n (%)  47s, n (%) | 386 (95.97) | 163 (97.02) | 223 (93.31) | **0.113** |
| >47, n (%) | 21 (4.03) | 5 (2.98) | 16 (6.69) |  |
| **Prothrombin time (PT), s** |  |  |  |  |
| ≤17, n (%) | 380 (93.37) | 156 (92.86) | 214 (89.54) | **0.252** |
| >17, n (%) | 37 (6.63) | 12 (7.14) | 25 (10.46) |  |
| **Trombin time (TT), s** |  |  |  |  |
| ≤19, n (%) | 393 (96.98) | 164 (97.62) | 229 (95.82) | **0.414*** |
| >19, n (%) | 14 (3.02) | 4 (2.38) | 10 (4.18) |  |
| **D-dimer, mg/L** |  |  |  |  |
| <0.5, n (%) | 181 (44.47) | 101 | 80 | <0.001 |
| ≥0.5, n (%)  0.5mg/L, n(%) | 226 (55.53) | 67 | 159 |  |
| **Fibrinogen (FIB), g/L** |  |  |  |  |
| <2, n (%) | 35 (8.60) | 12 | 23 | **0.380$, 0.050^** |
| 2-4, n (%) | 246 (60.44) | 113 | 133 |  |
| >4, n (%) | 126 (24.57) | 43 | 83 |  |
| **International normalized ratio (INR)** |  |  |  |  |
| ≤1.5, n (%) | 385 (95.47) | 162 (93.52) | 223 (95.81) | **0.170** |
| >1.5, n (%) | 22 (4.53) | 6 (6.48) | 16 (4.19) |  |
| **C-reactive protein, mg/L** | 10.30（2.08-49.15） |  |  |  |
| ≤10, n (%)  , n (%) | 152 (49.51) | 94 (40.51) | 58 (59.05) | <0.001 |
| >10/L, n (%) | 155 (50.49) | 60 (59.49) | 95 (42.95) |  |

* Fisher’s Exact test were used compare the COVID-19 mortality between the patients with different indices.

& Patients with or without comorbidities were compared.

@ Patients with or without CRUC were compared.

# COVID-19 mortality of patients with high neutrophil counts (>6.3×10⁹/L) or leukocyte counts（>10×10⁹/L) were compared with other two groups.

$ COVID-19 mortality of low platelet count group(<100×10⁹/L) and low FIB (<2g/L) were compared with the other two groups.

^ COVID-19 mortality of high platelet count group(>300×10⁹/L) and low FIB (>4g/L)were compared with the other two groups.
