# Supplementary material for: Elderly Male With Cardiovascular-Related Comorbidities Has a Higher Rate of Fatal Outcomes: A Retrospective Study in 602 Patients With Coronavirus Disease 2019
Source: Front Cardiovasc Med. 2021 Jun 7;8:680604. doi: 10.3389/fcvm.2021.680604 (PMC8215131; doi:10.3389/fcvm.2021.680604)
Supplement: Supplementary Table 3 — Baseline characteristics of male and female patients. *Chi-square tests or Fisher's exact test were used to compare the COVID-19 mortality between the patients with different indices. &Patients with or without underlying comorbidities were compared. @Patients with or without CRUC were compared. #COVID-19 mortality of patients with high neutrophil counts (>6.3 × 10^9^/l) or leukocyte counts (>10 × 109/l) was compared with the other two groups. $COVID-19 mortality of low-platelet-count group (<100 × 109/l) and low FIB (<2 g/l) was compared with the other two groups. ∧COVID-19 mortality of high-platelet-count group (>300 × 109/l) and low FIB (>4 g/l) was compared with the other two groups. [file Table_3.DOCX]

**Table S3. Baseline characteristics of male and female patients**

| Characteristics | All patients (n=602) | Male (n=313) | Female (n=289) | *P*-value |
| --- | --- | --- | --- | --- |
| **Demographic** |  |  |  |  |
| **Comorbidity** |  |  |  |  |
| No comorbidities, n (%) | 194 (33.44) | 95 (31.15) | 99 (35.87) | 0.228@, 0.273&, |
| CRUC, n (%) | 143 (24.66) | 75 (24.59) | 68 (24.64) |  |
| Other comorbidities, n (%) | 108 (18.62) | 57 (18.69) | 52 (18.84) |  |
| Two and more comorbidities, n (%) | 135 (23.28) | 78 (25.57) | 57 (20.35) |  |
| **Laboratory findings** |  |  |  |  |
| **Hematologic** |  |  |  |  |
| **Leukocyte count,10⁹ /L** |  |  |  |  |
| <4×10⁹ /L, n (%) | 95 (21.2) | 34 (12.19) | 61 (22.34) | 0.004*# |
| 4×10⁹/L-10×10⁹/L,n (%) | 284 (62.83) | 196 (70.25) | 188 (68.86) |  |
| >10×10⁹/L, n (%) | 73 (16.15) | 49 (17.56) | 24 (8.79) |  |
| **Neutrophil count,10⁹/L** |  |  |  |  |
| <1.8×10⁹ /L, n (%) | 33 (6.00) | 10 (3.58) | 23 (8.49) | <0.001*# |
| 1.8×10⁹/L-6.3×10⁹/L,n (%) | 384 (69.82) | 183 (65.59) | 201 (74.17) |  |
| >6.3×10⁹/L, n (%) | 133 (24.18) | 86 (30.83) | 47 (17.34) |  |
| **Lymphocyte count, 10⁹ /L** |  |  |  |  |
| <0.8×10⁹ /L, n (%) | 169 (30.73) | 104 (37.28) | 65 (23.99) | 0.001* |
| 0.8-4.0×10⁹ /L (%) | 381 (69.27) | 175 (62.7) | 206 (76.01) |  |
| **Platelet count, 10⁹ /L** |  |  |  |  |
| <100×10⁹ /L, n (%) | 25 (6.19) | 17 (7.98) | 8 (4.19) | **0.170$， 0.292^** |
| 100-300×10⁹ /L, n (%) | 295 (73.02) | 156 (73.04) | 139 (72.77) |  |
| >300×10⁹ /L, n (%) | 84 (20.79) | 40 (18.78) | 44 (23.04) |  |
| **Other indices** |  |  |  |  |
| **APTT, s** |  |  |  |  |
| ≤47s, n (%) | 386 (95.97) | 202 (93.52) | 184 (96.34) | **0.200** |
| >47s, n(%) | 21 (4.03) | 14 (6.48) | 7 (3.66) |  |
| **Prothrombin time(PT), s** |  |  |  |  |
| ≤17s, n (%) | 380 (93.37) | 189 (87.50) | 181 (94.76) | 0.018 |
| >17s, n(%) | 37 (6.63) | 27 (12.50) | 10 (5.24) |  |
| **Thrombin time (TT), s** |  |  |  |  |
| ≤19s, n (%) | 393 (96.98) | 207 (95.83) | 186 (97.38) | **0.392** |
| >19s, n(%) | 14 (3.02) | 9 (4.17) | 5 (2.62) |  |
| **D-dimer, mg/L** |  |  |  |  |
| <0.5mg/L, n (%) | 181 (44.47) | 78 (36.11) | 103 (53.93) | <0.001 |
| ≥0.5mg/L, n(%) | 226 (55.53) | 138 (73.89) | 88 (46.07) |  |
| **Fibrinogen (FIB), g/L** |  |  |  |  |
| <2, n (%) | 35 (8.60) | 19 (8.80) | 16 (8.38) | **1.000$, 0.012^** |
| 2-4, n(%) | 246 (60.44) | 118 (54.63) | 128 (67.02) |  |
| >4,n(%) | 126 (24.57) | 79 (36.57) | 47 (24.61) |  |
| **International normalized ratio (INR)** |  |  |  |  |
| ≤1.5, n (%) | 385 (95.47) | 202 (93.52) | 183 (95.81) | <0.001 |
| >1.5, n(%) | 22 (4.53) | 14 (6.48) | 8 (4.19) |  |
| **C-reactive protein, mg/L** | 10.30（2.08-49.15） |  |  |  |
| ≤10mg/L, n (%) | 152 (49.51) | 64 (40.51) | 88 (59.05) | 0.002 |
| >10mg/L, n(%) | 155 (50.49) | 94 (59.49) | 61 (42.95) |  |

* Chi-Square Tests or Fisher’s Exact test were used compare the COVID-19 mortality between the patients with different indices.

& Patients with or without underlying comorbidities were compared.

@ Patients with or without CRUC were compared.

# COVID-19 mortality of patients with high neutrophil counts (>6.3×10⁹/L) or leukocyte counts（>10×10⁹/L) were compared with other two groups.

$ COVID-19 mortality of low platelet count group(<100×10⁹/L) and low FIB (<2g/L)were compared with the other two groups.

^ COVID-19 mortality of high platelet count group(>300×10⁹/L) and low FIB (>4g/L)were compared with the other two groups.
